# Hyperinflammatory syndrome in a paediatric patient with a recent diagnosis of HIV/AIDS infection: hemophagocytic lymphohistiocytosis or immune reconstitution syndrome?

**DOI:** 10.1186/s12879-023-08457-9

**Published:** 2023-07-18

**Authors:** Fabrizio Leone, Nicola Cotugno, Chiara Casamento Tumeo, Paola Zangari, Patrizia Palomba, Rachele Adorisio, Fabrizio De Benedetti, Claudia Bracaglia, Paola Papoff, Camilla Ajassa, Paolo Palma, Stefania Bernardi

**Affiliations:** 1 Policlinico Umberto I, Maternal Infantile and Urological Sciences Department, Sapienza University of Rome, Rome, Italy; 2 Clinical and Research Unit of Clinical Immunology and Vaccinology, Academic Department of Pediatrics (DPUO), Bambino Gesù Children Hospital, IRCCS, Rome, Italy; 3 Chair of Pediatrics, Department of Systems Medicine, University of Rome “Tor Vergata”, Rome, Italy; 4 Academic Department of Pediatrics (DPUO), Bambino Gesù Pediatric Hospital, IRCCS, Rome, Italy; 5 Microbiology and Diagnostic Immunology Unit, Bambino Gesù Children’s Hospital, IRCCS, Rome, Italy; 6 Heart Failure, Transplant and Mechanical Cardiocirculatory Support Unit, Department of Pediatric Cardiology and Cardiac Surgery, Heart Lung Transplantation, ERN GUARD HEART: Bambino Gesù Hospital and Research Institute, Rome, Italy; 7 Division of Rheumatology, Bambino Gesù Children’s Hospital, IRCCS, Roma, Italy; 8 Department of Pediatrics, Pediatric Intensive Care Unit, Umberto I Policlinico, Sapienza University of Rome, Rome, Italy; 9 Department of Public Health and Infectious Diseases, Sapienza University of Rome, Rome, Italy; 10 Unit of Immune and Infectious Disease, University Department of Pediatrics, DPUO, Bambino Gesù Children’s Hospital, Rome, Italy

**Keywords:** HIV, AIDS, HLH, IRIS, Pediatric, Infection, Hyperinflammatory syndrome, MIS-C

## Abstract

**Introduction:**

Haemophagocytic lymphohistiocytosis is a rare and life-threatening condition caused by uncontrolled immune activation leading to excessive inflammation and tissue destruction. It could either be due to a primary genetic defect or be triggered by secondary causes such as infections, autoimmune diseases, rheumatological diseases or post-transplant immunosuppression. We here report the case of a 4-year-old child with a recent AIDS diagnosis who developed a severe systemic inflammation.

**Case report:**

We here report the case of a 4-year-old child with a recent AIDS diagnosis who was admitted to the ER with acute respiratory failure due to *Pneumocystis jiroveci* infection and Aspergillosis; the following microbiological assessment also showed a CMV, HSV, EBV and HHV-7 coinfection. On the 51st day after she’d started antiretroviral therapy, 39th after she’d followed a course of Bactrim and Caspofungin for PJI and Ambisome for pulmonary Aspergillosis, she started presenting fever, unresponsive to broad-spectrum antibiotic therapy. She also presented worsening of her clinical conditions, with evidence at the laboratory assessments of progressive raise in inflammatory indexes, coagulopathy, trilinear cytopenia and hyperferritinemia. To perform the differential diagnosis between IRIS and HLH, HLA-DR on T cells was studied, turning out negative for IRIS. Therefore, in the suspicion of HLH, a bone marrow aspirate and biopsy were performed with evidence of trilinear cytopenia, prevalence of T-cells and macrophages with signs of phagocytosis. She was started on high-dose steroids and Anakinra for a total of 29 days, resulting in prompt apyrexia and progressive improvement of her clinical conditions and laboratory results.

**Conclusion:**

To the best of our knowledge there is poor literature available about the differential diagnosis of HLH and IRIS, therefore medical management in the concurrence of these two conditions needs to be further investigated, especially in a setting where immunological testing is not quickly available. The clinical differences between these pathologies are blurred and the bone marrow biopsy within marker for IRIS helped us to distinguish these two entities.

## Background

Viral infections can trigger a systemic inflammatory response, which is necessary to contrast the spread and effects of the infection itself. In some pathological conditions, the systemic inflammatory response is not correctly counter-regulated and once it is triggered it cannot be normally contained and switched off. After COVID-19 pandemics we have witnessed the emergence of multisystem inflammatory syndrome in children (MIS-C), a rare post-infectious hyperinflammatory disorder associated with SARS-CoV-2 [1]. This disorder may also have overlapping features with Kawasaki disease (KD), hemophagocytic lymphohistiocytosis (HLH), and toxic shock syndrome (TSS). More in depth, primary (familial/hereditary) and secondary (non-familial/hereditary) hemophagocytic lymphohistiocytosis (HLH) are hyperinflammatory syndromes which can be triggered by a wide array of conditions. Secondary HLH (sHLH) includes infection- (e.g., viral/bacterial/fungal/parasitic) and non-infection-related diseases (e.g., autoimmunity or malignancy). Viral hemophagocytic lymphohistiocytosis (HLH) is the major type among all age groups: particularly Herpes Virus can trigger sHLH. Epstein-Barr virus (EBV)-related HLH (EBV-HLH) is the most common type of viral HLH in childhood [2]. The pathophysiology underlying HLH consists of an uncontrolled macrophage activation, resulting in a cytokine storm. Alongside NK cells and CD8^+^ lymphocytes are not able to counter regulate this process, which hesitates into an unbalanced hyperinflammatory process.

The diagnosis is made according to the criteria defined by the HLH-2004 [3] trial which includes either the confirmation of a mutation in an HLH gene or five out the following eight criteria (Table 1). To this date, these are the only available criteria for HLH and even if they were made for diagnosis of primary HLH are also used for the diagnosis of sHLH. The overall prognosis for HLH is poor, with high morbidity and mortality, the possibility of relapse, especially in cases with an underlying genetic defect. A prompt diagnosis and treatment are pivotal in determining the best outcome for the patients both in terms of morbidity and mortality [3, 4].

**Table 1:** Traditional HLH-2004 criteria [3] and Diagnostic Criteria for IRIS [10]

Traditional HLH-2004 criteria	Diagnostic Criteria for IRIS
At least five of the following	Major criteria
Fever > 38.5°C	
Splenomegaly	Atypical presentation of “Opportunistic infections of tumors” in patients responding to ART
Cytopenia affecting at least two cells lines	Decrease in plasma HIV RNA level by at least 1 log_10_copies/mL
Haemoglobin < 9 g/dL	
Platelets < 100 b/L	**Minor criteria**
Absolute Neutrophil Count < 1000 b/L	
Hypertriglyceridemia (fasting triglycerides > 265 mg/dL or > 3 mM and/or fibrinogen < 150 mg/dL	Increased blood CD4 + T-cell count after ART
Ferritin > 500 µg/L	Increase in immune response specific to the relevant pathogen, e.g. Delayed type hypersensitivity (DTH) response to mycobacterial antigens
Hemophagocytosis seen on tissue biopsy of bone marrow, spleen, lymph node, or liver	
Low/Absent NL-cell activity	
Soluble CD25 (soluble IL-2 receptor) > 2400 U/mL	

Immune Reconstitution Syndrome (IRIS) [5] following the initiation of antiretroviral therapy (ART) in patients with acquired immunodeficiency syndrome (AIDS), and Non-HIV IRIS [6] are two other hyperinflammatory syndromes that could mimic HLH. The differential diagnosis between the two conditions is hard to make clinically, as they present with similar symptoms; however, it can be made through an immunological laboratory assessment as IRIS is determined by the immunological rebound due to CD4-lymphocytes activation.

IRIS generally presents in two forms: “paradoxal IRIS” [7] refers to the worsening of the patient’s symptoms concerning a known coinfection, while “unmasking IRIS” [7] refers to the appearance of symptoms that may lead to “unmasking” of a not-previously-known coinfection. A low CD4^+^ cell count, a high antigen load from an opportunistic infection and a short time between ART initiation and coinfection treatment initiation are all risk factors for the development of IRIS [8, 9].

The diagnosis of IRIS can take place according to French et al. [10] by identifying two major and three minor criteria (Table 1). The clinical features (localized or systemic) of IRIS are related to the type and location of pre-existing opportunistic infection.

Specifically, CD4^+^ lymphocytes are usually reconstituted after ART has started in two phases: 3 to 6 months after ART initiation there is a rapid increase in CD4^+^, particularly CD45RO^+^ (memory T cells, they proliferate in response to recall antigen). Subsequently, there is an increase in CD45RA^+^ (naïve T cells) which expresses the expansion of T cell clones produced by the thymus. Along with the increase in T lymphocytes, there also is an increase in immune activation markers: HLA-DR^+^ and CD38^+^ on CD4 lymphocytes. Analysing these markers may help differentiate HLH and IRIS.

Making the correct diagnosis is critical as HLH requires specific treatment (e.g. immunosuppressants) and carries a worse prognosis, while IRIS could only require, in its mild form, support therapy with nonsteroidal anti-inflammatory agents and other supportive drugs. We here report the case of a 4-year-old child with a recent AIDS diagnosis who develop a severe systemic inflammation.

## Case presentation

**Fig. 1:**
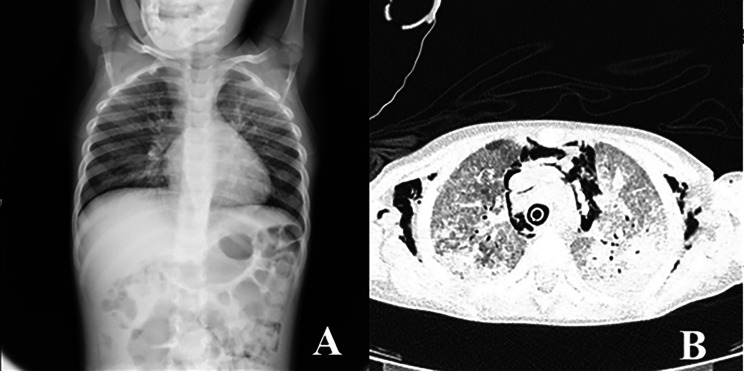
Chest X-Ray (**A**) and CT (**B**) showing multiple and bilateral ground glass areas and patchy consolidations in the inferior lobes, pneumomediastinum

A 4-year-old female Caucasian child was admitted to the emergency department with fever and acute respiratory failure. The personal and familial anamnestic recall brought no elements of suspicion for a past SARS-CoV-2 infection. The chest X-ray and subsequent computed tomography (CT) showed multiple and bilateral ground glass areas and patchy consolidations in the inferior lobes, pneumomediastinum with supraclavicular and cervical bilateral subcutaneous emphysema (Fig. 1). The microbiological assessment on broncho-alveolar lavage (BAL) was positive for *Pneumocystis jiroveci* (PJ) and galactomannan, SARS-CoV-2 proved negative. As her respiratory dynamics progressively deteriorated, she was intubated and assisted through mechanical ventilation.

At the anamnestic recall the parents reported a history of recurrent respiratory infections since she was 3 years old, a previous episode of ocular HSV infection and recurrent oral thrush. Due to the patient’s medical history and the evidence of PJI and pulmonary aspergillosis, an immunological assessment was performed, and a severe CD4-penia emerged: CD4^+^ was 1.06% (6 cell/µl, normal value 500–1000). Soon after the diagnosis of HIV-positivity was finalised with a viral load of 83.429 copies/ml. She was classified as a stage 3, according to Centres for Disease Control (CDC) classification system for HIV infection [11]. Combined ART was initiated at the diagnosis of HIV infection, with a lamivudine, zidovudine and lopinavir/ritonavir; alongside treatment for PJ and aspergillosis was started with Trimethoprim/Sulfamethoxazole, Caspofungin and Ambisome.

The microbiological assessment run to investigate possible coinfections proved positivity for CMV (31,446 copies/ml) and EBV (8542 copies/mL). Also, at the oral cavity inspection, the patient presented some vesicles positive for HSV. Acyclovir and Gancyclovir were then added to her therapeutic regimen. On the 51st day after she had started ART, she started presenting fever with a progressive worsening of clinical conditions: no other microbiological agents were isolated at the analysed samples (blood, stools and urine) and there was no improvement with broad-spectrum antibiotic therapy.

**Fig. 2:**
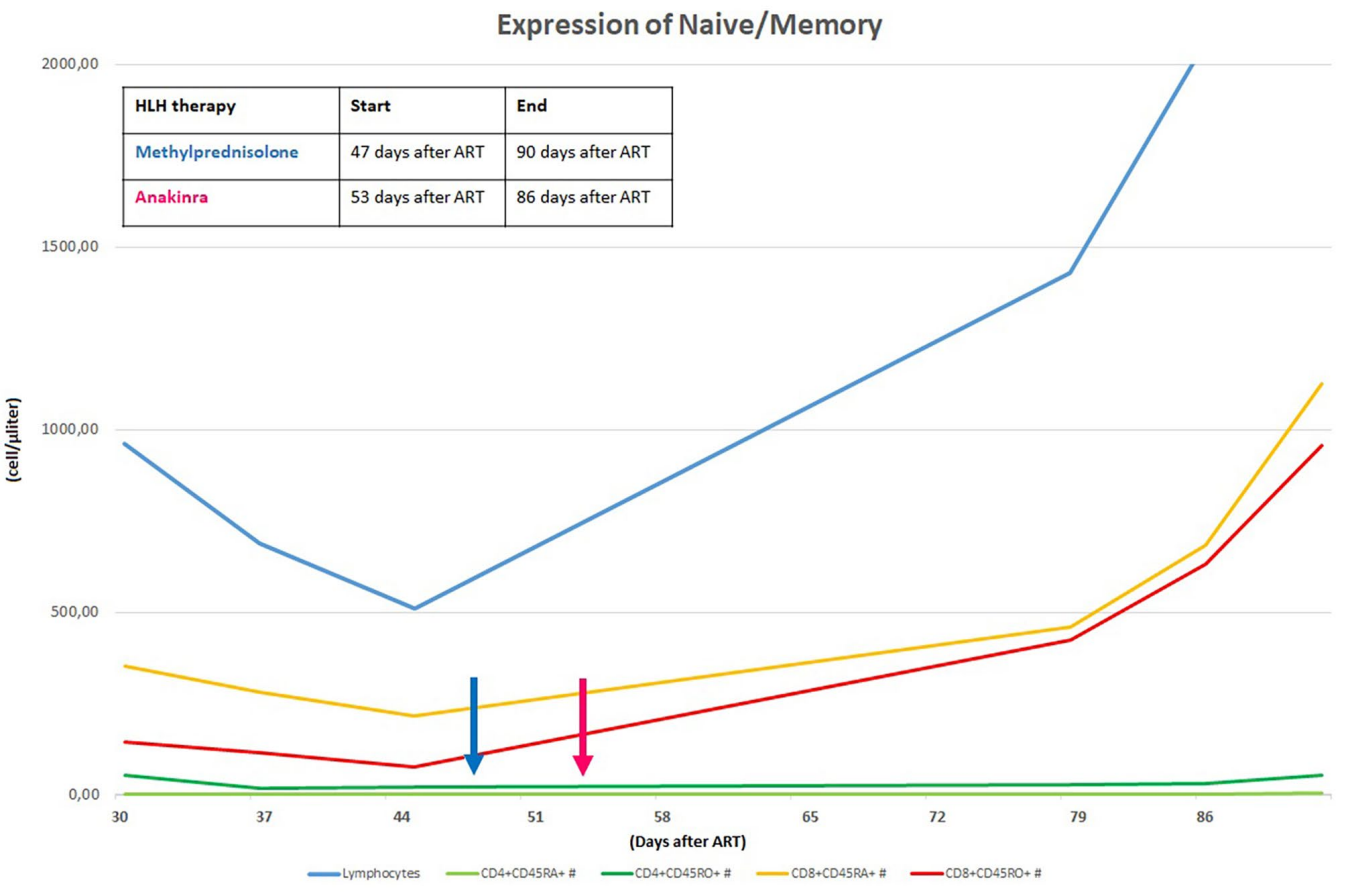
CD4^+^ and CD8^+^ RA/RO progression after ART initiation

Her laboratory assessment showed progressive trilinear cytopenia (lowest values: haemoglobin 7,7 g/dl, absolute neutrophil count 690 cells/mcl, platelet count 14.000 cells/mcl), progressive increase of C-reactive protein (up to 4,28 mg/dl), hyponatremia (serum sodium 129 mEq/l), hypoalbuminemia (3,1 g/dl) and hypofibrinogenemia (76 mg/dl ). Triglycerides were slightly increased (160 mg/dl) and ferritin levels were increased (up to > 12.000 ng/ml). Cardiac enzymes showed progressive elevation (high sensitivity troponin up to 40,9 pg/ml and proBNP 1548 pg/ml). At that time her HIV viral load was undetectable and CD4^+^ cell count was 35 cells/mcl (normal value 630–2110). We have always studied the expansion trend of expansion of CD4^+^ cell in relation to CD8^+^ cell, we also evaluating the expression of CD45 RA^+^RO^−^ (naïve) and CD45RA^−^RO^+^ (memory) on the T cells: these analyses were compatible with the success of ART (Fig. 2).

**Fig. 3:**
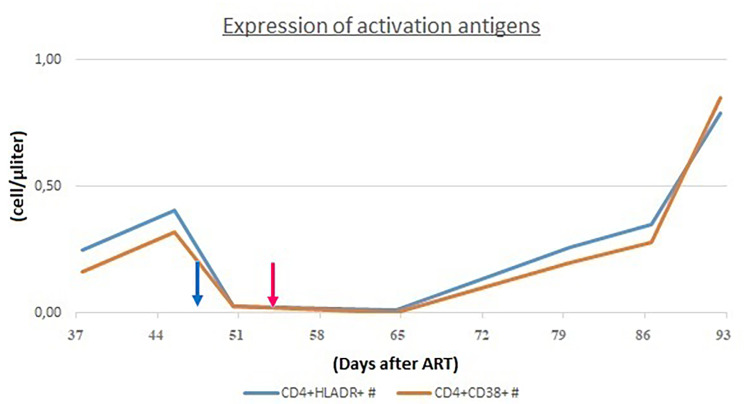
CD4^+^ cells did not show immune activation markers (HLADR^+^ and CD38^+^)

**Fig. 4:**
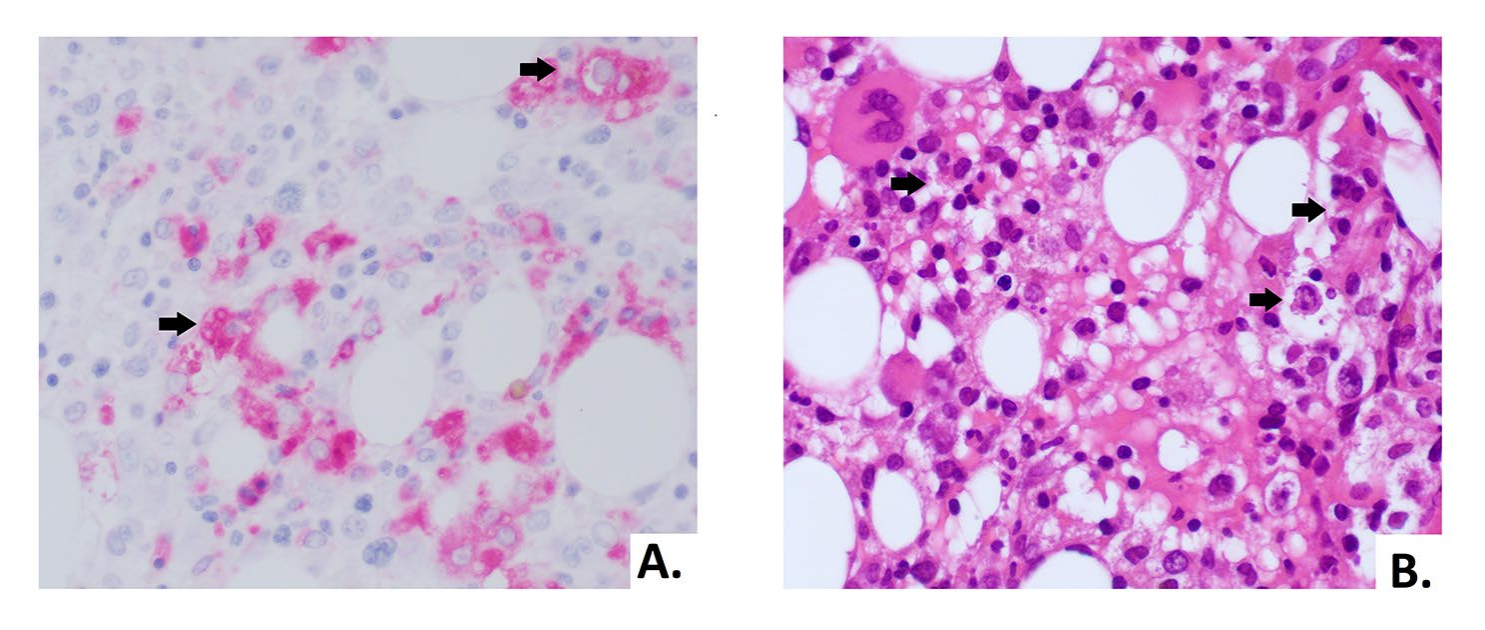
The biopsy showed a bone marrow with low cellularity, marked reduction of erythroid line, predominantly T lymphocytosis and histiocytosis with aspects of hemophagocytosis (arrows). **(A)** Morphology of histiocytes in CD68 immunohistochemical staining (40X). **(B)** Hematoxylin and eosin stain of bone marrow biopsy with hemophagocytosis (40X)

**Fig. 5:**
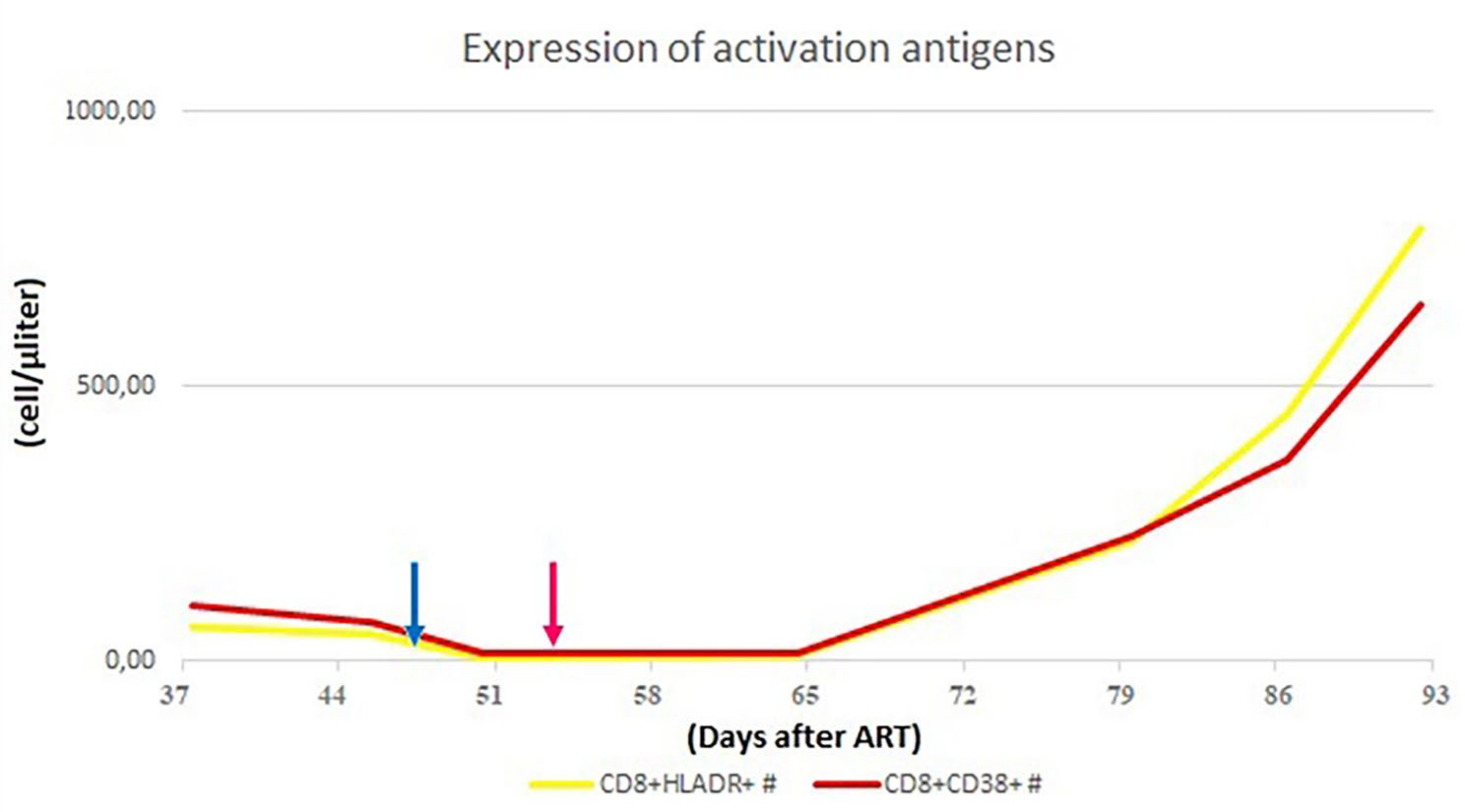
CD8^+^ cells showed immune activation markers (HLADR^+^ and CD38^+^)

In order to assess the differential diagnosis between HLH and IRIS, T-cell activation was investigated through the HLA-DR^+^ and CD38^+^ evaluation on CD4^+^ lymphocytes, which resulted always less than 1/microliter (Fig. 3). In the suspicion of HLH and in order to assess other causes of cytopenia, a bone marrow aspirate and biopsy were performed: evidence of bone marrow cytopenia (Fig. 4) with prevalence of T-cells and macrophages with signs of phagocytosis was found. The immune activation markers, HLA-DR + and CD38+, were present on the CD8^+^ lymphocytes (Fig. 5), making the diagnosis of HLH even more suggestive [12]. We could not assess soluble IL2-R at that time in our hospital.

**Fig. 6:**
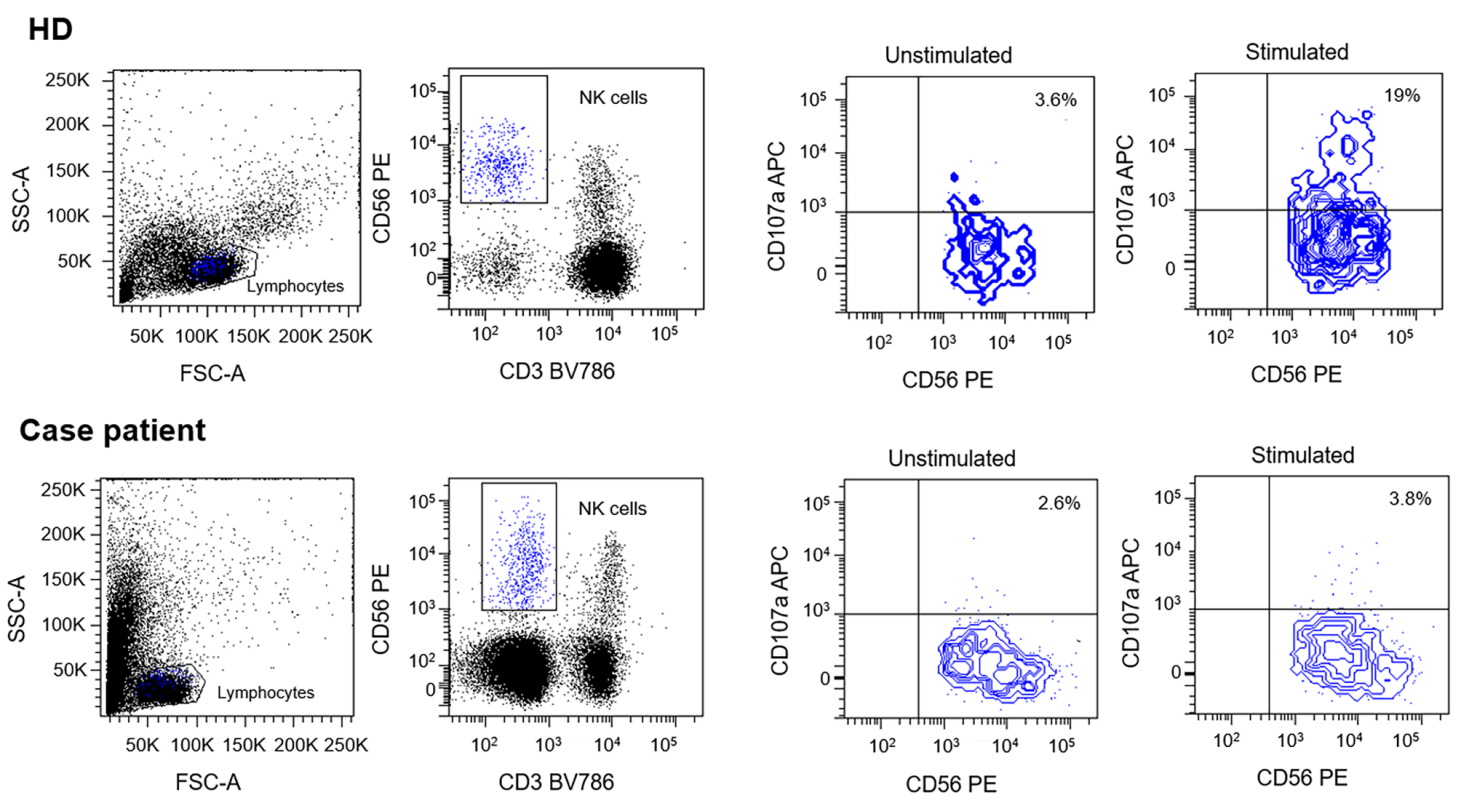
In order to evaluate the expression of CD107a and its relative effect on NK cells degranulation, Healthy Donor (HD) and patients’-derived Peripheral Blood Mononuclear Cells (PBMC) were isolated from EDTA-blood samples. Specimens were diluted in Phosphate Buffered Saline (PBS) solution and layered over Ficoll 1.077 g/ml. Then, 2 × 106 cells/cm2 were plated on cell culture dishes in RPMI medium containing 50 U/ml penicillin, 50 mg/ml streptomycin, 2 mM L-glutamine and 10% Fetal Bovine Serum (FBS). Human IL-2 (100U/mL) was added to allow cells expansion. Cells were incubated overnight at 37 °C. The day after, PBMC were co-cultured with or without lymphoblast K562 target cells at E:T ratio 1:1 for 3 h at 37° C. Cells were then stained with anti-human CD3, CD56 and CD107a antibodies. Results were evaluated by comparing CD107a expression between PBMC with or without co-cultured K562 cells. Data were acquired with a FACS LSRFortessa (Becton Dickinson, USA). Flow cytometer profiles were analyzed using FACSDiva Software (Becton Dickinson, USA). Patient’s NK showed no degranulation after stimulation with K562

Due to the presence of six diagnostic criteria [3] the diagnosis of HLH was made: persistent fever > 38,5 °C, cytopenia involving more than two lineages, hypertriglyceridemia and hypofibrinogenemia, splenomegaly, hyperferritinemia and hemophagocytosis in bone marrow. Furthermore, Patient’s NK showed a lower degranulation after stimulation with K562 cells than healthy donor (Fig. 6). However due to an ongoing treatment with systemic corticosteroids such assay can only be partially considered reliable for degranulation.

Then a glucocorticoid therapy with three pulses of methylprednisolone (30 mg/kg/day) was started. The patient returned upyretic after the first pulse of methylprednisolone. After the three pulses, she was started on dexamethasone (10 mg/m^2^/day) as maintenance therapy. The laboratory assessment showed a progressive improve of the inflammatory parameters with worsening cytopenia and coagulopathy.

In consideration of the insufficient response to glucocorticoid therapy, the treatment was implemented with intravenous interleukin-1 receptor antagonist (Anakinra, 100 mg twice a day = 14 mg/kg/day).

After the treatment with anakinra was started, the patient’s clinical conditions and laboratory parameters showed a progressive improvement. Glucocorticoid therapy was progressively reduced and the interleukin-1 receptor antagonist was initially reduced to 7 mg/kg/dose (100 mg once per day) after 21 days of treatment. Anakinra was reduced by 25% after 5 days; after 3 days, the dose was reduced by 30% and eventually stopped after an additional 24 h of treatment. After the immunosuppressive therapy was stopped, the patient maintained good clinical condition and normalization of inflammatory markers.

## Discussion and conclusions

A prompt diagnosis of HLH is crucial for defining the clinical outcome.

In the reported case, the patient had a clear hyperinflammatory response as showed by laboratory parameters (progressive cytopenia, coagulopathy, hyperferritinemia and high inflammatory markers) and clinical course (persistent fever, deterioration of clinical conditions, splenomegaly). Though she presented with some of the most commonly IRIS-associated coinfections (such as PJ and EBV), she did not present a fully compatible clinical picture. The immunological assessment made in part possible to differentiate the two conditions as markers for IRIS (HLA-DR^+^ and CD38^+^ on CD4^+^ lymphocytes) were negative. Bone marrow biopsy confirmed the suspicion of HLH. Once the diagnosis was made, immunosuppressive treatment was started. Traditionally the HLH-94 protocol defines the specifical therapy for HLH, consisting of 8 weeks of induction therapy with etoposide and dexamethasone [4]. A wide array of protocols has been developed ever since, such as the HLH-2004 protocol which implements the HLH-94 protocol with cyclosporine alongside etoposide and dexamethasone, and more traditional chemotherapy regimens like CHOP (cyclophosphamide, doxorubicin, vincristine and prednisone) and DEP (doxorubicin, etoposide and methylprednisolone). For these different protocols no studies of superiority to HLH-94 have been finalised to this date.

Treatment for secondary HLH is suppressing hyperinflammation, targeted therapy with anti-IL1 directed therapies (e.g. anakinra) or antitumor necrosis factor alpha-directed agents (anti- α TNF) have started being used, but only few case reports exist to this date [13]. However in children, Anakinra has been shown to be effective in secondary sHLH [14]. Other lines of treatment are commonly considered for classical HLH children such as interferon gamma (IFNγ)-blocking antibody (Emapalumab) [15] or anti CD-52 (alemtuzumab) [16], however, they are most commonly used lines in primary HLH or relapsed post-hematopoietic stem cell transplantation (HSCT). Considering the underlying chronic HIV infection and the severe immune compromission at the time of HLH onset, we opted for an alternative treatment such as anakinra and methylprednisolone.

The therapeutic approach for IRIS, instead, would have been based upon the treatment of the underlying coinfection and control of the immune hyperinflammatory response with supportive therapy [17].

The complex therapeutic decision to treat with immunosuppressant drugs an immunocompromised patient is largely discussed by Fazal et al. [18]. The authors reported two adult HIV infected patients with HLH treated with a different therapeutic approach and fatal outcome. In the review done by Tabaja et al. [19] 78% of the HIV infected patients had at least 1 infectious agent - other than HIV – that probably trigger HLH. The most common agents were EBV (26%), HHV-8 (21%) and Histoplasma capsulatum (17%) and the survival rate was 60%. Among those, 93% received treatment for identified secondary triggers, while 51% received HLH-directed therapy. There was significant heterogeneity in the choice regimens for HLH. In the review by Nguyen et al. [20] Histoplasma seemed to be the most common cause behind HLH cases in HIV patients and treating promptly the underlying infection improved the patients’ clinical outcome. However, this infection was not revealed in our patient.

To the best of our knowledge there is poor literature available about the differential diagnosis of HLH and IRIS, therefore medical management in the concurrence of these two conditions needs to be further investigated, especially in a setting where immunological testing is not quickly available.

The clinical differences between these pathologies are blurred and diagnostic criteria are not always diriment. Heterozygous mutations of familial HLH genes (e.g. PRF1, UNC13D, STX11, STXBP2, LYST, RAB27A, AP3B1) could be observed in over 40% of individuals suffering from sHLH [21]. A whole exome sequencing has not been performed in our patient and this represents a limitation of our case report description; however, this aspect should be assessed in patients with sHLH. An additional limitation of the present case is that markers suggestive for pHLH such as NK degranulation were only partially reliable at the moment of blood sampling since the patient was already under systemic corticosteroids. Additional studies further exploring this clinical suspect have been planned and will be performed on the patient. In conclusion, this case suggests that bone marrow biopsy in association with markers for IRIS seem to be crucial in distinguishing HLH and IRIS in a patient living with HIV with a systemic inflammatory syndrome.

## Data Availability

All data generated or analysed during this study are included in this published article.

## List of abbreviations

AIDS: Acquired ImmunoDeficiency Syndrome
ART: AntiRetroviral Therapy
CHOP: cyclophosphamide, doxorubicin, vincristine and prednisone
COVID-19: COronaVIrus Disease 19
DEP: doxorubicin, etoposide and methylprednisolone
EBV: Epstein-Barr Virus
EBV-HLH: EBV-related HLH
HLH: Hemophagocytic LymphoHistiocytosis
HSTC: Hematopoietic Stem Cell Transplantation
sHLH: Secondary HLH
HIV: Human Immunodeficiency Virus
IFNγ: interferon gamma
IRIS: Immune Reconstitution Syndrome
KD: Kawasaki Disease
MISC: Multisystem Inflammatory Syndrome in Children
TSS: Toxic Shock Syndrome
